# The “beauty premium” effect of voice attractiveness of long speech sounds in outcome-evaluation event-related potentials in a trust game

**DOI:** 10.3389/fpsyg.2022.1010457

**Published:** 2022-10-13

**Authors:** Junchen Shang, Zhihui Liu

**Affiliations:** ^1^Department of Medical Humanities, School of Humanities, Southeast University, Nanjing, China; ^2^School of Psychology, Liaoning Normal University, Dalian, China

**Keywords:** voice attractiveness, duration, trust game, cooperative behavior, the “beauty premium” effect

## Abstract

Previous research suggested that people with attractive voices had an advantage in economic games, even if the voices were only presented for 400 ms. The present study investigated the influence of voice attractiveness on the cooperative trust behavior with longer exposure times to the voices. Event-related potentials (ERPs) were recorded during the feedback outcome evaluation. Participants heard a voice of the partner for 2,040 ms and decided whether to invest to the partner for a possibility to gain more money. The results showed that participants made more invest choices to the attractive partners, replicating the “beauty premium” effect of the attractive voices. Moreover, participants were more likely to invest to male partners. The ERP analysis for the outcome showed that the difference waves of feedback-related negativity (FRN) amplitude were smaller in the attractive voice condition than in the unattractive voice condition, suggesting that the rewarding effect of attractive voices weakened the frustrating feelings of the loss. In sum, the present study confirms that attractive voices with longer presentation durations facilitate cooperative behavior and modulate the processing of feedback evaluations.

## Introduction

With the development of Internet communication technology, people use voice more often for communication with social network software, which can improve work efficiency. If you want to contact someone, you can hear the person’s voice first not only by a telephone call but also by mobile apps. Face-to-face communication is eliminated, making social communication more convenient. The social signals conveyed by voices have important impact on daily life. Many mobile apps such as navigation software have begun to use voice for human–computer interaction. Human voice is applied more and more often in the media. The “sound industry” is booming, such as the Chinese TV show “The Sound,” audiobooks, and radio apps. In literature, there are also depictions of personality traits conveyed by human voice. In “A Dream of the Red Mansions,” one of the most famous literature in China, Wang Xifeng’s hearty laughter and unfettered speech showed her shrewd, strong, pungent, and vicious traits even if she did not show up. Voice attractiveness is the extent to which the voice of the speaker can induce a positive and pleasant emotional experience and attract other people. Zuckerman and Driver (1989) revealed the “what sounds beautiful is good” stereotype, such that voice attractiveness influenced impressions of personality (Zuckerman et al., 1990). A recent study (Wu et al., 2021) confirmed that voice attractiveness was related to the speaker’s personality, such as capability and approachability dimensions in Chinese culture. Voice attractiveness also plays an important role in evolution because it correlates with traits reflecting hormone levels and health (Groyecka et al., 2017). In addition to behavioral research, some studies provided evidence for the neural underpinnings of voice attractiveness processing with functional magnetic resonance imaging (fMRI) and event-related potentials (ERPs). When participants passively listened to voices in a pure tone detection task, the activities in the higher level auditory cortex and inferior prefrontal regions were correlated with voice attractiveness (Bestelmeyer et al., 2012). Moreover, compared to happiness and age judgments of voices, voice attractiveness judgments activated the bilateral inferior parietal cortex, and the dorsomedial prefrontal cortex extending into the perigenual anterior cingulate cortex (Hensel et al., 2015). An ERP study (Zhang et al., 2020) reported that attractive voices elicited larger N1, smaller P2, and larger P3 and late positive component (LPC) amplitudes than unattractive voices in an attractiveness rating task. Since attractive faces also evoked larger LPC than unattractive faces (Ma and Hu, 2015; Ma et al., 2015, 2017), and dorsomedial prefrontal cortex was also involved in facial attractiveness processing (Hensel et al., 2015), voice attractiveness may have a reward effect as facial attractiveness (Shang and Liu, 2022).

Research revealed that people who own attractive voices had some advantages in economic activities. A recent study (Shang et al., 2021) suggested that males’ voice attractiveness affected responders’ fairness considerations during the ultimatum game even though the voices were only presented for 400 ms. More offers were accepted from proposers who had attractive voices in a two-person ultimatum game. Moreover, voice attractiveness of the third player also influenced decision-making in a three-person ultimatum game which included a proposer, a responder, and a powerless third player. Participants (responders) accepted more offers if the third player had an attractive voice even though the offer was unfair for them but fair for the third player. The above findings confirmed that voice attractiveness induced the “beauty premium” effect. Shang and Liu (2022) further explored the influence of vocal attractiveness on cooperative behavior in a trust game using similar voice stimuli as Shang et al. (2021). The participants made more invest choices to the partners with attractive voices. However, vocal attractiveness did not impact the feedback-related negativity (FRN) related to the outcome which is an important component in the economic decision-making.

Despite previous research showing the influence of voice attractiveness on decision-making in ultimatum game and trust game (Shang et al., 2021; Shang and Liu, 2022), the exposure time to the voices in these studies is only 400 ms. Research showed that increased exposure time resulted in more differentiated trait inferences of an unfamiliar face although people can form impressions (such as attractiveness) even after a 100-ms exposure time (Willis and Todorov, 2006). Moreover, the judgment of facial attractiveness correlates with the judgment of voice attractiveness (Saxton et al., 2009; Hughes and Miller, 2016). It is possible that “beauty premium” effect would be different between long speech voices and short speech voices. In addition, voices last for much longer time in daily life. Some studies (Krumpholz et al., 2021, 2022) suggested that it took around 1 s for the stable judgment for voice attractiveness. This duration is much longer than the exposure time to the voices in decision-making research (Shang et al., 2021; Shang and Liu, 2022). However, it is unclear whether decision-making toward voices with exposure time longer than 1 s would be different with previous studies (Shang et al., 2021; Shang and Liu, 2022).

Especially, the FRN amplitude, which represents the brain sensitivity to the failure of decision-making, has been well documented (e.g., Xu et al., 2020a,b). It is considered as the brain response to positive and negative outcomes, such as gain and loss (Gehring and Willoughby, 2002; Yeung and Sanfey, 2004; Martín, 2012; Ma and Hu, 2015; Ma et al., 2017). For example, unfair offers elicited larger FRN than fair offers when the partner’s face was unattractive in the Ultimatum Game, whereas there was no difference in attractive-face condition (Ma et al., 2015, 2017). It is possible that voice attractiveness would induce the same effect in trust game. The reason that Shang and Liu (2022) did not find the influence of vocal attractiveness on FRN may be because of the short duration of voices. Therefore, the present study investigated the influence of voice attractiveness of long speech voices (lasted for 2,040 ms) on the investment behavior and the neural underpinnings for outcome evaluation in a trust game (Shang and Liu, 2022) using ERPs. The present study predicted that participants would invest more money to the attractive partners. It is also predicted that the FRN effect in the attractive voice condition would be different with that in the unattractive voice condition.

## Materials and methods

### Participants

Using G* Power v. 3.1.9.6 (Faul et al., 2007), the sample size was determined based on the sample size of previous ERP research about voice attractiveness and trust game (Shang and Liu, 2022). Given the power of a statistical test of 0.95, and the effect size of 0.25, 64 students (33 female participants, *M*_*age*_ = 20.94 years, *SD* = 2.79 years) at Liaoning Normal University participated in this study. The participants all had normal or corrected to normal vision and normal hearing. All participants were physically healthy and had no neurological damage. Each participant was paid a certain money reward after the experiment. For this research, we obtained approval from the ethics committee of Liaoning Normal University and written informed consent from each participant before the experiment.

### Design and materials

This experiment employed a within-subject design with voice attractiveness (attractive vs. unattractive) and voice gender (female participant vs. male participant) as within-subject factors. The ratio of investment and the ERP amplitudes (FRN) were the dependent variables.

To be comparable with Shang and Liu (2022), the neutral vowels were used. Voice stimuli were chosen from Ferdenzi et al. (2015). There were 111 voice samples (61 female voices, 50 male voices, *M*_*age*_ = 22.9 years, *SD* = 4.3 years). Each voice sample included three neutral vowel syllables (/i/,/a/,/ou/). The duration of all voice recordings is adjusted to 2,040 ms, by using Praat software v.5.3.85. The sound intensity is adjusted to 70 dB. Fifty-eight participants (18 male participants, *M*_*age*_ = 21.60 years, *SD* = 2.43 years) who did not take part in the ERP experiment were asked to rate the attractiveness of the voices on a seven-point Likert scale (from 1 = “very unattractive” to 7 = “very attractive”).

According to the mean rating value of each voice across the 58 participants, we chose 30 female voices (15 most attractive voices and 15 most unattractive voices) and 30 male voices (15 most attractive voices and 15 most unattractive voices) for use as partners in the trust game for the ERP experiment. The attractiveness ratings of the four categories of voices were compared using a two-way ANOVA. The voice attractiveness was significantly different (*F*_(1, 56)_ = 362.55, *p* < 0.001, η_*p*_^2^ = 0.87, 95%CI [0.79, 0.90]). The main effect of voice gender was not significant [*F*_(1, 56)_ = 0.40, *p* = 0.531]. The interaction between voice attractiveness and voice gender was not significant [*F*_(1, 56)_ = 2.15, *p* = 0.148]. The attractive female voices (*M* = 5.15, *SD* = 0.31) were rated as more attractive than unattractive female voices (*M* = 2.81, *SD* = 0.49). The attractive male voices (*M* = 4.91, *SD* = 0.38) were rated as more attractive than unattractive male voices (*M* = 2.91, *SD* = 0.55).

The acoustic parameters of attractive voices and unattractive voices were calculated using Praat software and were compared using paired *t*-tests (as shown in Tables 1, 2). Previous research suggested that lower-pitched male voices are more attractive than higher-pitched male voices (Collins, 2000; Feinberg et al., 2005; Jones et al., 2010; Re et al., 2012). Also, higher-pitched female voices are more attractive than lower-pitched female voices (Feinberg et al., 2008; Zheng et al., 2020). Almost consistent with prior studies, differences in voice attractiveness for the present experiment were accompanied by differences in acoustic parameters. F0 of unattractive male voices was higher than attractive male voices. Moreover, f3 of attractive male voices was higher than unattractive male voices. F0 and f4 of unattractive female voices were lower than attractive female voices. In attractive and unattractive condition, F0, f3, f4, Df, Pf, and HNR of female voices were higher than male voices. In attractive condition, the jitter of female voices was lower than male voices. In addition, the shimmer was lower in female voices.

**TABLE 1:** Means (and standard deviations) and acoustic differences between attractive and unattractive voices.

	Female voices					Male voices				
	Attractive voices	Unattractive voices	*t*	*p*	Cohen’s *d*	Attractive voices	Unattractive voices	*t*	*p*	Cohen’s *d*
F0	252.51 (18.69)	229.72 (31.44)	2.41	0.023	0.88	132.65 (15.50)	151.02 (25.77)	–2.37	0.025	–0.86
f1	747.77 (58.11)	701.19 (97.84)	1.59	0.124	0.58	712.56 (107.99)	653.57 (78.97)	1.71	0.099	0.62
f2	1,781.70(79.23)	1,756.65(105.50)	0.74	0.468	0.27	1,783.02(121.58)	1,727.10(78.07)	1.50	0.145	0.55
f3	3,055.14(97.62)	3,032.83(92.25)	0.64	0.525	0.24	2,982.77(90.17)	2,900.92(101.09)	2.34	0.027	0.86
f4	4,192.94(73.43)	4,078.56(130.68)	2.96	0.006	1.08	3,981.34(171.73)	3,945.07(119.19)	0.67	0.507	0.25
Df	1,148.39(33.06)	1,125.79(31.98)	1.90	0.067	0.70	1,089.60(40.10)	1,097.17(32.55)	–0.57	0.575	–0.21
Pf	0.54 (0.44)	0.12 (0.83)	1.75	0.091	0.64	−0.05(0.92)	−0.60(0.63)	1.91	0.067	0.70
Jitter	1.94 (0.60)	1.87 (0.79)	0.28	0.782	0.10	2.55 (0.82)	2.37 (0.68)	0.66	0.516	0.24
Shimmer	7.47 (1.33)	8.41 (2.48)	–1.29	0.207	–0.47	10.82 (3.47)	10.84 (2.20)	–0.02	0.981	–0.01
HNR	14.05 (2.28)	13.87 (3.38)	0.18	0.862	0.06	10.00 (2.03)	11.07 (1.46)	–1.66	0.109	–0.61

F0, fundamental frequency in Hz; f1–f4, formant frequencies in Hz; Df, formant dispersion in Hz; Pf, formant position; Jitter, variation of pitch in μs; Shimmer, variation of energy in dB; HNR, harmonic-to-noise ratio in dB.

**TABLE 2:** Means (and standard deviations) and acoustic differences between female and male voices.

	Attractive voices					Unattractive voices				
	Female voices	Male voices	*t*	*p*	Cohen’s *d*	Female voices	Male voices	*t*	*p*	Cohen’s *d*
F0	252.51 (18.69)	132.65 (15.50)	19.12	<0.001	6.98	229.72 (31.44)	151.02 (25.77)	7.50	<0.001	2.74
f1	747.77 (58.11)	712.56 (107.99)	1.11	0.276	0.41	701.19 (97.84)	653.57 (78.97)	1.47	0.154	0.54
f2	1,781.70(79.23)	1,783.02(121.58)	–0.04	0.972	–0.01	1,756.65(105.50)	1,727.10(78.07)	0.87	0.391	0.32
f3	3,055.14(97.62)	2,982.77(90.17)	2.11	0.044	0.77	3,032.83(92.25)	2,900.92(101.09)	3.73	<0.001	1.36
f4	4,192.94(73.43)	3,981.34(171.73)	4.39	<0.001	1.60	4,078.56(130.68)	3,945.07(119.19)	2.92	0.007	1.07
Df	1,148.39(33.06)	1,089.60(40.10)	4.38	<0.001	1.60	1,125.79(31.98)	1,097.17(32.55)	2.43	0.022	0.89
Pf	0.54 (0.44)	−0.05(0.92)	2.26	0.032	0.83	0.12 (0.83)	−0.60(0.63)	2.67	0.012	0.98
Jitter	1.94 (0.60)	2.55 (0.82)	–2.32	0.028	–0.85	1.87 (0.79)	2.37 (0.68)	–1.87	0.072	–0.68
Shimmer	7.47 (1.33)	10.82 (3.47)	–3.50	0.002	–1.28	8.41 (2.48)	10.84 (2.20)	–2.85	0.008	–1.04
HNR	14.05 (2.28)	10.00 (2.03)	5.15	<0.001	1.88	13.87 (3.38)	11.07 (1.46)	2.95	0.006	1.08

F0, fundamental frequency in Hz; f1–f4, formant frequencies in Hz; Df, formant dispersion in Hz; Pf, formant position; Jitter, variation of pitch in μs; Shimmer, variation of energy in dB; HNR, harmonic-to-noise ratio in dB.

### Procedure

Participants comfortably completed the experiment individually in a sound-attenuated lab. A chin rest was used to eliminate head movements. The voices were presented binaurally over Sennheiser headphones. Before the experiment, we adjusted the loudness for each participant for the comfortableness. The instructions and measurements were controlled by E-prime version 2.

**FIGURE 1:**
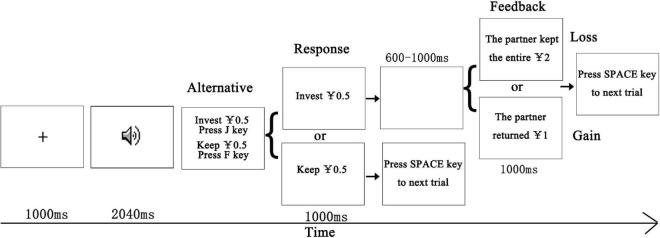
Trial procedure of the trust game task. A central fixation cross was presented followed by a partner’s voice presented for 2,040 ms. The participant was asked to decide whether to invest ¥0.5 or refuse to invest ¥0.5. The decision would be presented for 1,000 ms. If the participant decided to invest, the partner would gain ¥2. Then, the partner either gave ¥1 back to the participant or kept the entire ¥2. If the participant refused to invest ¥0.5, the amount of money would remain unchanged.

This experiment employed the same trust game in Shang and Liu (2022), except for the voice samples and duration of voices (see Figure 1). We clarify the procedure succinctly. In the beginning, there were eight practice trials containing the voices which were not shown in the formal experiment. First, participants got ¥20 to play the game. They were asked to decide whether to invest to a “real” partner (who was actually fictional and represented by an attractive or an unattractive voice) in each trial for a chance to earn the real monetary remuneration as the final rewards they gained in the game. In each trial, a central fixation cross was first shown for 1,000 ms. Then, a voice of the partner was presented for 2,040 ms. Afterward, two sentences “invest ¥0.5” and “keep ¥0.5” appeared on the screen. Half of the participants pressed the “F” key once they decided to invest and pressed the “J” key once they decided to keep ¥0.5. For the other half of participants, the response keys were counterbalanced. If the participants submitted the choice, the final decision would be shown for 1,000 ms. If the participant chose to invest, a blank screen was shown for 600–1,000 ms, and the partner would receive ¥2. The partner would either pay ¥1 to the participant or keep all of the rewards. Then, the feedback from the partner was presented for 1,000 ms. If participants refuse to invest, the current amount of money would not be changed. Finally, participants were asked to press the space key to start the next trial. Each voice was repeated eight times. Half times the voice was accompanied by gains, while the other half times it was accompanied by losses. The ERP experiment contained 480 trials presented in a pseudorandom order, whereas the participants were not told about the regularity.

### Event-related potential recording and analysis

The electroencephalography (EEG) was continuously recorded from 64 scalp sites arranged on an elastic cap (Brain Products, GmbH, Germany). The sampling rate was 500 Hz. The ground electrode was on the cephalic (forehead) location. The vertical and horizontal electrooculogram (EOG) were recorded with two electrodes which were placed below and on the right side of the right eye. All electrode impedances were kept below 5 kΩ. The EEG signals were re-referenced offline to the average of the left and right mastoids. First, the EOG artifacts were corrected. Then, digital bandpass filtering was employed between 0.01 and 30 Hz. We applied an independent component analysis algorithm to correct EOG. Epochs, which contained EOG artifacts and amplifier clipping artifacts, were excluded before averaging. Other recording artifacts were also excluded when the EEG amplitudes exceeded ±80 μV. The ERPs were extracted and segmented with time-locked signal averaging by adopting the time window initiated at −200 ms and stopped at 1,000 ms relative to the feedback stimuli onset.

The average amplitude of FRN differential waves (280–310 ms) was measured to investigate the ERP waves evoked by feedback stimuli in “investment” trials. Based on the methodology in previous research (Holroyd and Krigolson, 2007; Chen et al., 2012), the difference waves of FRN amplitude were calculated by subtracting the average amplitude of the gain ERP wave from the average amplitude of the loss ERP wave. Five electrode sites (Fz, FCz, Cz, CPz, and Pz) were selected. The FRN difference waves were separately calculated for two conditions: attractive voice-related FRN difference wave and unattractive voice-related FRN difference wave. We did not examine the voice gender effect since there were not enough artifact-free trials in each condition [we used criteria of Shang and Liu (2022) that at least 30 valid trials per condition]. Six participants were excluded, and there were 58 valid participants (32 female participants) in the analysis of FRN differential waves. A two-way repeated measures ANOVA was conducted on FRN differential amplitudes including voice attractiveness (attractive vs. unattractive) and electrode sites (Fz, FCz, Cz, CPz, and Pz) as within-subject factors. We adopted Greenhouse–Geisser corrections when the results violated the spherical assumption. All multiple comparisons were Bonferroni-corrected.

## Results

### Behavioral results

We calculated the percentage of average percentage of invest choices in attractive and unattractive voice conditions, respectively, as the ratio of investment. We conducted a 2 (voice attractiveness: attractive vs. unattractive) × 2 (voice gender: female participant vs. male participant) repeated measures ANOVA on the ratio of investment.

This test yielded a significant effect of voice attractiveness (*F*_(1, 63)_ = 63.47, *p* < 0.001, η_p_^2^ = 0.50, 95%CI [0.32, 0.62]). Participants were more willing to invest to attractive partners (*M* = 0.66, *SD* = 0.14) than unattractive partners (*M* = 0.55, *SD* = 0.15). There was also a significant effect of voice gender (*F*_(1, 63)_ = 9.62, *p* = 0.003, η_p_^2^ = 0.13, 95%CI [0.02, 0.29]), indicating that participants were more likely to cooperate with male partners (*M* = 0.63, *SD* = 0.13) than female partners (*M* = 0.58, *SD* = 0.16). The interaction between voice attractiveness and voice gender was not significant [*F*_(1, 63)_ = 0.15, *p* = 0.698].

### Event-related potential results: The feedback-related negativity difference wave (280–310 ms)

**FIGURE 2:**
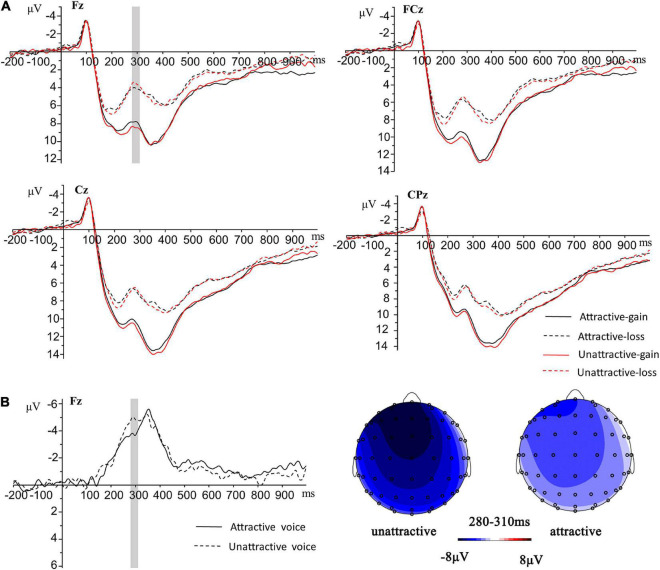
**(A)** Grand average event-related potentials (ERPs) induced by feedback of gain and loss at four representative electrodes in the attractive and unattractive voice conditions. **(B)** Topography of scalp distribution and differential waves generated by gain and loss in the attractive and unattractive voice conditions.

The results showed a significant effect of voice attractiveness (*F*_(1, 57)_ = 4.33, *p* = 0.042, η_p_^2^ = 0.07, 95%CI [0.00, 0.22]). Specifically, a larger difference wave of FRN amplitude was elicited by unattractive voices than attractive voices. The main effect of electrode sites was significant (*F*_(1.39, 79.25)_ = 13.97, *p* < 0.001, η_p_^2^ = 0.20, 95%CI [0.06, 0.34]). *Post-hoc* comparisons showed that a smaller FRN difference wave was elicited in parietal region than the other regions (*p*s < 0.003). A larger FRN difference wave was elicited in fronto-central and central regions rather than central-parietal region (*p*s ≤ 0.006). The interaction between voice attractiveness and electrode sites was not significant [*F*_(1.67, 94.88)_ = 0.50, *p* = 0.576] (Figure 2).

## Discussion

The current research investigated the effect of voice attractiveness on investments in a trust game when the duration of partner’s voice was 2,040 ms. The behavioral responses suggested that participants made more invest choices in the attractive partner condition than in the unattractive partner condition. The finding was in line with prior studies which revealed the “beauty premium” effect of voice attractiveness in the ultimatum games and a trust game using short voices which lasted for 400 ms (Shang et al., 2021; Shang and Liu, 2022). The result was also similar with the “beauty premium” effect of facial attractiveness in decision-making (e.g., Ma et al., 2015, 2017). Hensel et al. (2015) suggested that the dorsomedial prefrontal cortex which was activated by voice attractiveness also played an important role in processing of facial attractiveness, indicating that the brain regions related to voice attractiveness overlapped with those activated by facial attractiveness. The present study confirmed this by behavioral findings.

We also analyzed participants’ decisions toward male partners and female partners. Specifically, participants made more cooperation choices (investments) to male partners compared to female partners. These results were inconsistent with previous research (Shang and Liu, 2022) which reported that people made more investments to female partners compared to male partners when the voices were unattractive. A possible explanation might be that objective and subjective judgments of voice traits can be different between short speech sounds and longer speech sounds (Pisanski and Feinberg, 2018; Krumpholz et al., 2022). Shang and Liu (2022) used short vowels, which may convey a first impression (Krumpholz et al., 2022). It is possible that gender influenced first impression of voice attractiveness. In the current study, each voice consisted of three vowels and lasted longer than 1 s. This duration enabled stable voice judgment (Krumpholz et al., 2021). Thus, the discrepancy of gender effect may be attributed to the exposure time of voices. Furthermore, the findings of present research supported previous research on facial attractiveness and decision-making, reporting that people allocated more money to male partners in an ultimatum game (Solnick and Schweitzer, 1999). This study further indicates that decision-making may be influenced by the gender of the partner with whom we interact even though only a voice was presented.

In addition, we analyzed participants’ brain activities in outcome feedback evaluations. We assumed that Shang and Liu (2022) did not yield the FRN effect because of the short exposure times to voices. Consistent with our hypothesis, the present research showed that the different waves of FRN amplitude (loss ERP minus gain ERP elicited by the feedback) in the unattractive voice condition were larger than in the attractive voice condition. This may be interpreted as a reward effect of voice attractiveness, which could affect participants’ fairness considerations and reduce their negative emotion toward loss even though the attractive partners did not return the reward. The findings also supported the beauty premium effect that participants may show more prosocial behaviors to partners with attractive voices (Shang et al., 2021). A similar FRN effect was reported by previous research about facial attractiveness and decision-making (Ma et al., 2017), such that unfair offers elicited larger FRN than fair offers in the unattractive face condition during an ultimatum game. Research showed that increased exposure time to a face may boost confidence in impressions (Willis and Todorov, 2006). The discrepancy of FRN observed in the present study compared with Shang and Liu (2022) might also be interpreted as a boosted confidence in voice attractiveness judgments after a longer exposure time, since impressions of voice attractiveness correlated with impressions of facial attractiveness (Saxton et al., 2009; Hughes and Miller, 2016). Again, the present study provides more evidence for the beauty premium of voice attractiveness of longer speech sounds in a social economic game.

There were two limitations in the current study. First, the attractiveness ratings of voices were from 58 participants (18 male participants) and were mainly based on female participants. Although the gender of participants was approximately balanced in the ERP experiment, the effect of attractiveness may be influenced by the biased ratings in the pretest selection. Second, we used long neutral vowel stimuli to rule out irrelevant variables, such as semantic meaning (Ferdenzi et al., 2013). However, the vowel sounds were not representative in everyday life and less ecologically. The raters’ evaluations of vowels may be different with words and speech in a real-life situation (Ferdenzi et al., 2013; Pisanski and Feinberg, 2018). Future research should test the beauty premium effect of voices using real speech sounds in natural social conditions.

## Conclusion

The present study suggested that both voice attractiveness and gender influenced investments in a trust game. Attractive voices facilitated cooperative behaviors, demonstrating the “beauty premium” effect. Participants were more likely to cooperate with male partners. Regarding the evaluation of feedback, larger FRN effects were observed in the unattractive voice condition than in the attractive voice condition, suggesting that the level of reward expectation may be higher in the unattractive partner condition.

## Data availability statement

The raw data supporting the conclusions of this article will be made available by the authors, without undue reservation.

## Ethics statement

The studies involving human participants were reviewed and approved by the Institutional Review Board of the Liaoning Normal University, China. The participants provided their written informed consent to participate in this study.

## Author contributions

JS designed the experiment. ZL prepared the materials and performed the experiments. Both authors analyzed the data and wrote the manuscript.
